# Adverse events recording in electronic health record systems in primary care

**DOI:** 10.1186/s12911-017-0565-7

**Published:** 2017-12-06

**Authors:** Sabine E. M. de Hoon, Karin Hek, Liset van Dijk, Robert A. Verheij

**Affiliations:** NIVEL, Netherlands Institute for Health Services Research, P.O. Box 1568, 3500 BN Utrecht, The Netherlands

**Keywords:** Recorded medication adverse events, Between practice variation, General practice, Patient safety

## Abstract

**Background:**

Adequate record keeping of medication adverse events in electronic health records systems is important for patient safety. Events that remain unrecorded cannot be communicated from one health professional to another. In the absence of a gold standard, we investigate the variation between Dutch general practices in the extent to which they record medication adverse events.

**Methods:**

Data were derived from electronic health records (EHR) of Dutch general practices participating in NIVEL Primary Care Database (NIVEL-PCD) in 2014, including 308 general practices with a total practice population of 1,256,049 listed patients. Medication adverse events were defined as recorded ICPC-code A85 (adverse effect medical agent). Between practice variation was studied using multilevel logistic regression analysis corrected for age, gender, number of different medicines prescriptions and number of chronic diseases.

**Results:**

In 2014 there were 8330 patients with at least one medication adverse event recorded. This corresponds to 6.9 medication adverse events per 1000 patients and is higher for women, elderly, patients with polypharmacy and for patients with comorbidity. Corrected for these patient characteristics the median odds ratio (MOR = 1.92) suggests an almost twofold difference between general practices in recorded medication adverse events.

**Conclusion:**

Our results suggest that improvement in terms of uniformity in recording medication adverse events is possible, preventing potential damage for patients. We suggest that creating a learning health system by individual practice feedback on the number of recordings of adverse events would help practitioners to improve their recording habits.

## Background

Medication adverse events such as opioid-induced constipation and myalgia caused by statins, are important patient safety indicators and a priority topic according to the World Health Organization [1, 2]. In this paper a medication adverse event is defined as a response to a drug which is noxious and unintended, and which occurs at doses normally used in man for the prophylaxis, diagnosis, or therapy of disease, or for the modification of physiological function [3]. A recent review demonstrates a bidirectional causal link between medication adverse events and non-adherence to medication therapy [4]. Adverse events can therefore negatively affect the pharmacological treatment effect which can eventually lead to hospital admissions.

Patient awareness and notification of relevant medication adverse events as well as adequate patient-communication when medicines are prescribed can improve patient safety. A report of the Institute of Medicine states that poor exchange of medical information and communication between healthcare professionals are responsible for medication adverse events and harm patient safety [5]. Safety research in other industries, such as aviation, also show that lack of communication and teamwork, and miscommunication are factors that contribute to adverse events [6]. Adequate recording of medication adverse events in healthcare can help overcome this since recorded events can be communicated. Furthermore, it is a prerequisite for creating a learning health system, both at the level of society as well as at the level of health care practices.

Most patient safety research in healthcare focuses on hospitalized patients. However, in many countries primary care is the first point of contact between patients and the healthcare system. As such, most of the care is provided there and the general practitioner has a large share in the medication that is prescribed. Because of the gatekeeper role, general practitioners (GPs) play an important part in patient safety by adequately signaling and recognizing medication adverse events [2, 7, 8]. Subsequently, uniform recording these events in patient’s electronic health record (EHR) is important to monitor progress and to ensure that all responsible parties are aware of this possible safety hazard [9]. Routine EHRs can play an important role in achieving a learning health system [10], improving healthcare. At patient level, EHRs play a key role in communication since events that remain unrecorded will not be communicated. This communication is crucial when multiple health care providers are involved in patient care, especially since patient care becomes more complex [8, 11].

Other countries with a comparable primary care structure, such as the UK, already showed that EHR data can be used to identify medication adverse events [12] alongside for example national incident reporting systems. In this paper we investigate the extent to which medication adverse events are routinely recorded in Dutch general practice and the variation between practices. We control for patient characteristics that are assumed to be strongly associated with the actual occurrence of adverse events. We control for age as it is known that older patients experience more medication adverse events [12, 13]. Other patient characteristics such as gender, polypharmacy, and comorbidity are also included, as they have found to be risk factors for medication adverse events as well [13, 14]. Underlying assumption in this paper is that the larger part of the remaining between practice variation does not represent differences in the *actual occurrence* of adverse events, but differences in the *recording* of these events.

## Methods

### Study population

Data were derived from EHRs of Dutch general practices that participated in NIVEL Primary Care Database (NIVEL-PCD) in 2014 [15]. Like in several other European countries (e.g. UK, Denmark), GPs in the Netherlands have a fixed population list that can change every three months. Only general practices with complete data (at least 46 weeks of recording) were included. Patients with missing data on gender (*n* = 12) and age (*n* = 13) were excluded from the analyses and we could use data from 308 general practices with a total listed population of 1,256,024 individuals.

### Measures

Symptoms and diagnoses are recorded routinely in general practice by practice nurses as well as GPs using the International Classification of Primary Care (ICPC) [16]. Medication adverse events should be recorded as patient contacts with ICPC-code A85 (adverse effect medical agent) in the EHR. Medication adverse events were calculated as the number of patients with at least one contact or episode of medication adverse event per 1000 listed patients. Medicines prescribed are recorded according to the Anatomical Therapeutic Chemical (ATC) classification system. The number of different medicines (unique ATC-codes) prescribed to an individual patient in 2014 was used as an indicator for polypharmacy and categorized in four categories (0, 1–4, 5–9 and ≥10 different medicines per patient). The number of chronic diseases in each individual patient was counted and categorized in four categories (0, 1, 2, and >2 chronic diseases per patient).

### Statistical analysis

To study between practice variation in recorded medication adverse events, multilevel logistic regression was performed adjusting for patient characteristics. In this multilevel logistic model patients (first level) were nested within general practices (second level). We first fitted an empty model (model 0) which allowed us to detect a possible general practice effect. We then estimated a model including patient age (centered to the mean) and gender (model 1) followed by a model including the number of different medicines prescribed to a patient (model 2) and number of chronic diseases in this patient (model 3). Differences between software packages in completeness of recording have been shown in other electronic health record based studies [17]. There may be differences between software brands in the extent to which they help the general practitioner to recognize, acknowledge and record adverse events. Analyses of differences between software package brands showed considerable variation in recorded adverse events, ranging from 5.1 to 10.4 adverse events per 1000 listed patients, dependent on the software brand. Therefore, all models were adjusted for software package used by the general practice. Odds ratio’s (OR), 95% confidence intervals (95%-CI), and *p*-values were calculated. To describe between practice variation in recorded medication adverse events, we calculated practice variance components, median odds ratio’s (MOR) and intraclass coefficients (ICC) [18]. The practice variance component indicates the variance between practices. The ICC indicates the proportion of variance that is attributable to differences between general practices. In this study, MOR refers to the increased probability of a recorded medication adverse event between two randomly chosen practices. Furthermore, we reported the probability of medication adverse event recorded and the 95%-CI around this mean per general practice. In order to make these results more interpretable, all determinants were centered to their mean with the most common software package as reference. All analyses were conducted using STATA version 14.0.

### Privacy

The study was carried out in accordance with Dutch legislation on privacy. According to Dutch legislation, neither obtaining informed consent nor approval by a medical ethics committee is obligatory for observational studies [19]. This study has been approved by the applicable governance bodies of NIVEL Primary Care Database under number NZR00315.055.

## Results

### Medication adverse events

Of the 1,256,024 patients aged between 0 and 112 years the average age was 40.7 years. The age and gender distribution of the patients in this study corresponds with the general Dutch population. As can be found in Table 1, 68.0% of the patients received different medicines and 38.7% had at least one chronic disease. This is comparable to the general Dutch population. There were 8330 patients with a least one recorded medication adverse event. This corresponds to 6.9 medication adverse events per 1000 listed patients. Recorded medication adverse events are twofold higher in women compared to men (9.4 and 4.3 per 1000 listed patients respectively) and gradually increases with age, except for 5–17 year olds having lower rates than younger children. Similarly, the number of recorded medication adverse events increases with the number of medicines prescribed and with the number of chronic diseases (Table 2).

**Table 1:** Characteristics of 1,256,049 patients in 308 general practices in the study

	Study population n (%)
Gender	
Male	620,000 (49.4)
Female	636,024 (50.6)
Age	
0–4	64,080 (5.1)
5–17	188,189 (15.0)
18–44	429,686 (34.2)
45–64	353,843 (28.2)
65–74	125,886 (10.0)
75–84	68,175 (5.4)
85+	26,165 (2.1)
Number of different medicines prescribed	
0	401,783 (32.0)
1–4	559,774 (44.6)
5–9	201,738 (16.1)
≥ 10	92,731 (7.4)
Number of chronic diseases	
0	769,604 (61.3)
1	280,156 (22.3)
2	111,195 (8.9)
> 2	95,069 (7.6)

**Table 2:** Number of patients with medication adverse events recorded, by patient group

	N	Per 1000 patients
Gender		
Male	2584	4.3
Female	5746	9.4
Age		
0–4	132	2.3
5–17	269	1.5
18–44	2085	5.1
45–64	2321	6.7
65–74	1671	13.5
75–84	1315	19.9
85+	537	21.9
Number of different medicines prescribed		
0	148	0.4
1–4	2174	4.0
5–9	2888	14.5
≥ 10	3120	34.4
Number of chronic diseases		
0	2289	3.1
1	2039	7.5
2	1564	14.4
> 2	2438	26.5

### Between practice variation

**Fig. 1:**
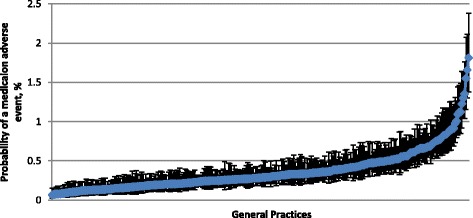
Adjusted probability of a medication adverse event recorded in a patients’ EHR, per practice. Adjusted for age, gender, number of different medicines and software package (model 2). Variables were centered around their mean and the most common software package served as reference. Each dot represents a general practice. The error bars represent the 95% confidence interval around the estimate of that practice

Table 3 presents the results of the models analyzing the probability of recording a medication adverse event using ICPC-code A85. The empty model shows the probability that a general practice records a medication adverse event as A85 is 0.93% (95%-CI: 0.24–3.55%). The between practice variance was 0.48 (standard errors (SE) = 0.05) which corresponds to an ICC of 0.129. This means that 12.9% of the variability in recorded medication adverse events can be attributed to differences between practices (and not individuals). When taking into account patient characteristics, similar practice variances with corresponding ICCs are observed. Practice variance is 0.47 (SE = 0.05) when adjusted for age and gender (model 1) and 0.46 (SE = 0.05) when number of different medicines was added to this model (model 2). This suggests that variability in recorded medication adverse events can still be attributed to between practices differences. Due to the high correlation between number of different medicines prescribed and chronic diseases (*r* = 0.58), adding the latter to this model did not improve the model (practice variance of 0.46, ICC = 12.2%, and MOR = 1.91). The number of medicines prescribed was most strongly associated with medication adverse events. The median odds ratio calculated from the practice variances is fairly high in the empty model (MOR = 1.94) and remains equally high after adjustment (MOR = 1.92). This means that after accounting for patient characteristics, the probability of a recorded medication adverse event is still almost two times higher in one practice compared to another. This variability is also presented in Fig. 1.

**Table 3:** OR and 95%-CI for a medication adverse event recorded for an patient within a practice

	Empty model	Model 1^a^	Model 2^b^
*Fixed effects*			
Patient characteristics			
Age		1.03 (1.03–1.03)^c^	1.00 (1.00–1.01)
Gender			
Male		1	1
Female		2.07 (1.97–2.17)	1.64 (1.57–1.72)
Number of different medicines prescribed			
0			1
1–4			9.93 (8.40–11.73)
5–9			34.74 (29.37–41.09)
≥10			81.22 (68.49–96.33)
*General effects at practice level*			
Practice variance (SE)	0.48 (0.05)	0.47 (0.05)	0.46 (0.05)
MOR	1.94	1.92	1.92
ICC	0.129	0.124	0.124

*SE* standard error, *MOR* Median Odds Ratio, *ICC* intraclass correlation coefficient

^a^model 1 adjusted for age and gender, ^b^model 2 adjusted for age, gender, number of different medicines prescribed. All models were adjusted for software package used by the general practice

^c^represented as Odds Ratio (95% confidence interval)

## Discussion

We investigated the variation in frequency of recording of medication adverse events between general practices in the Netherlands and to what extent this variation can be attributed to differences between practices or patients within the practices. We found that on average 6.9 medication adverse events are recorded per 1000 patients. General practices differ considerably in recording medication adverse events, with a median odds ratio of 1.94. As expected, more medication adverse events were found in elderly, patients with polypharmacy and chronic diseases. Other studies confirm these findings [14, 20, 21]. After accounting for these patient characteristics, a considerable amount of between practice variation in recorded medication adverse events remains. The median odds ratio still shows almost a twofold difference when comparing two random general practices, with 12.4% of the variability attributed to the level of practices.

Other studies examining medication adverse events in primary care focus on person-years or number of consultations as the unit of analysis. In a supplementary analysis we found that 2.3 consultations (*n* = 11,004) per 1000 consultations with the general practice concerned a recorded medication adverse event. Number of contacts were calculated on the basis of insurance claims codes representing (telephone) consultations and home visits. In an observational study using an English general practice research database Tsang et al. showed a slightly lower overall incidence of 6.0 medication adverse events per 1000 person-years, and 8.0 adverse events per 10,000 consultations [14]. Another study in English general practices based on routinely recorded data found 1.26 adverse events per 1000 consultations [12]. Compared with these studies, the overall incidence of recordings of medication adverse events seems to be relatively low. Whether this is due to differences in recording or real incidence remains to be investigated.

This study is one of the first studies to investigate between practice variation in medication adverse event recordings. It could be that this process can be facilitated by the software package used in the general practice. Differences between software packages in completeness of recording have been shown in other electronic health record based studies [17], as the software package also seems to affect the quality of prescribing [22]. Optimizing the electronic health record system can help the general practitioner to detect and record adverse events. We also found considerable variation in recorded adverse events between six different software packages. All models were subsequently adjusted for software package. However, between practice variation remained suggesting that other practice characteristics such as age or years of experience of the general practitioner can explain this variation. Also social and socio-economic difference between general practices and migration background would be relevant additional demographic characteristics to explain remaining variation. Within the scope of this paper it was not possible to explore these issues further.

One possible explanation for the between practice variation found in this study may be that it represents the actual ‘true’ variation between practice populations. However, given the fact that we controlled for the most important individual patient level factors of medication adverse events (i.e. age, gender, polypharmacy, and chronic diseases), we believe other explanations may be more important. Another possible explanation is that doctors differ in the extent to which they ‘recognize’ adverse events. Another possibility is that medication adverse events are assigned to other ICPC codes by some GPs. Rather than the diagnosis code ‘adverse effect medical agent’ (ICPC code A85), general practitioners may also use ICPC code A13 which indicates the symptom ‘concern of medical agent’. However, sensitivity analysis showed that ICPC code A13 and A85 are only modestly correlated at practice level (correlation coefficient = 0.4) suggesting that A85 represents medication adverse events as diagnosed by the general practitioner. Yet another possibility is that general practitioners may also record medication adverse events as a symptom presented by patients. For example myalgia may have been recorded as an adverse event (ICPC code A85) related to the use of statins and as myalgia (ICPC code L18) by another. Yet another explanation may be that some general practitioners use free text to record medication adverse events instead of the required fields. A qualitative study exploring above mentioned possible explanations can help to better understand the variation in recorded events, but this was not possible within the scope of this paper. Moreover, because of the important role pharmacists play in medication management of patients, it is possible that part of the unexplained between practice variation is due to factors related to the (relation between GP practice and) pharmacy. It could be that patients discuss medication adverse events with their pharmacist (for example when they collect their medicines). If this information is not subsequently communicated with the GP, it will not be recorded in the GPs electronic health records data. Our analysis on the role of software packages showed that GP software packages that have strong links with the information systems used by pharmacies, recorded most medication adverse events. It include the pharmacist-related factors when exploring between practice variation concerning medication.

Whatever the explanation, the relatively large amount of between practice variation found in this study strongly suggests that adverse event recording in electronic health records systems is far from uniform. Uniformity is essential for adequate exchange of information between health professionals and is important with respect to patient safety [6]. Studies looking at incident reporting systems in other industries refer to meaningful feedback information as an important aspect to improve safety. Other industries also tell us that rather than simply providing feedback a learning culture it is also required to attain a successful safe organization [23–25]. Also in primary healthcare research it has been found that providing data quality feedback and benchmark information to general practices reduces between practice variation by making practice personnel aware of the variation and of their recording habits and stimulating them to adhere to recording guidelines [17]. This learning health system approach would not only enhance uniformity of recording but also uniformity in identifying or recognizing adverse events.

## Conclusions

This study shows that on average 6.9 medication adverse event are recorded per 1000 patients in general practice. This figure is higher for women, elderly and patients with polypharmacy and comorbidity. However, after accounting for these patient characteristics, variation in recorded medication adverse events still shows large differences between general practices. This suggests that improvement in terms of uniformity of recording medication adverse events is possible. To improve this situation, we suggest that practices should be made aware of these differences, using practice feedback and benchmarking tools, as part of a larger scheme to create a learning culture.

## Abbreviations

ATC: Anatomical Therapeutic Chemical
CI: Confidence Interval
EHR: Electronic Health Record
GP: General practitioner
ICC: Intraclass Coefficient
ICPC: International Classification of Primary Care
MOR: Median Odds Ratio
OR: Odds ratio
SE: Standard Error
